# Association between Mindfulness and Weight Status in a General Population from the NutriNet-Santé Study

**DOI:** 10.1371/journal.pone.0127447

**Published:** 2015-06-03

**Authors:** Géraldine M. Camilleri, Caroline Méjean, France Bellisle, Serge Hercberg, Sandrine Péneau

**Affiliations:** 1 Université Paris 13, Equipe de Recherche en Epidémiologie Nutritionnelle, Centre de Recherche en Epidémiologie et Statistiques, Inserm (U1153), Inra (U1125), Cnam, COMUE Sorbonne Paris Cité, Bobigny, France; 2 Unité de Surveillance et d’Epidémiologie Nutritionnelle, Institut de Veille Sanitaire, Université Paris 13 Sorbonne Paris Cité, Bobigny, France; 3 Département de Santé Publique, Hôpital Avicenne, Bobigny Cedex, France; University of Bath, UNITED KINGDOM

## Abstract

**Background:**

Mindfulness is defined as non-judgmental awareness of the present moment. There is some evidence of the efficacy of mindfulness-based interventions in weight loss. However, this psychological concept has only been rarely explored in observational studies, and no study to date has examined the association between dispositional mindfulness and weight status in a large population-based sample.

**Objective:**

We aimed to examine the relationship between mindfulness scores and weight status in a large sample of the adult general population in France.

**Design and Methods:**

A total of 14,400 men and 49,228 women aged ≥18 y participating in the NutriNet-Santé study were included in this cross-sectional analysis. We collected mindfulness data using the Five Facet Mindfulness Questionnaire as well as self-reported weight and height. The association between weight status and dispositional mindfulness, as well as its subscales (observing, describing, acting with awareness, non-judging and non-reactivity), was assessed using multinomial logistic regression models adjusted for socio-demographic and lifestyle factors.

**Results:**

Women with higher dispositional mindfulness scores were less likely to be overweight (excluding obesity) (OR quartile 4 vs. 1 = 0.84, 95% CI: 0.79-0.90) and obese (OR quartile 4 vs. 1 = 0.71, 95% CI: 0.65-0.78). In addition, overall, in this group, all subscales were inversely associated with weight status, with the strongest association found for the “observing” subscale. In men, higher mindfulness was associated only with lower odds of obesity (OR quartile 4 vs. 1 = 0.81 (0.69, 0.96)), and only the “observing” and “non-reactivity” subscales were significantly inversely associated with weight status.

**Conclusion:**

Results support the interest of a shift in perspective that takes into account positive psychological and cognitive factors such as dispositional mindfulness in the investigation of obesity and its associated factors.

## Introduction

Overweight and obesity have reached epidemic proportions worldwide and represent a major global health burden in light of their numerous co-morbidities, i.e. cardiovascular disease, diabetes and cancer [1]. Psychological and cognitive processes have a strong influence on dietary intake. For instance, reduced mealtime attention to what one eats, due to distraction or lack of visual information on the amount of food consumed, has been shown to increase immediate intake and possibly later intake as well [2]. In observational studies, the likelihood of overweight or obesity increased with the frequency of eating while performing another activity, such as watching TV [3].

Increasing awareness of food and of the eating process might be an effective alternative to restrictive diets [2,4] that have little effect upon long-term weight loss [5,6]. Mindfulness can be defined as the awareness that emerges through paying attention on purpose, in the present moment, and nonjudgmentally to the unfolding experience [7]. This set of skills is innate, reflected in a general tendency to be mindful in daily life, but can also be developed via meditation and relaxation exercises [7,8].

Dispositional mindfulness has rarely been examined in epidemiological studies. The few observational studies showed contrasting results, with a negative association between dispositional mindfulness and weight gain [9] or else no overall significant differences in anthropometric measurements between less mindful and more mindful groups [10]. Few observational studies have specifically addressed mindful eating, which can be defined as non-judgmental awareness of physical and emotional sensations associated with eating [11,12]. Those studies showed a negative association with BMI. However, previous studies were carried out on samples limited either by small sample size or by the lack of demographic heterogeneity (students, military recruits and women), and most of them did not take into account potential confounding factors that could influence both mindfulness and weight. Large population-based studies are needed to clarify these associations.

Mindfulness meditation was first introduced into medicine and health care as a complement to medical treatment to help patients cope with stress, pain and disability [7]. The literature supports the usefulness of mindfulness-based stress reduction programs for a broad range of chronic disorders in stress-related outcomes [13,14]. Mindfulness-based interventions have been recently extended to the treatment of obesity and related eating behaviors [15,16]. Several mindfulness-based interventions reported positive but overall small effects on body weight, i.e., weight maintenance [17] and weight loss [18,19], among overweight/obese participants. Other studies presented non-significant results [20,21].

Thus far, the role of sex in the association between dispositional mindfulness and weight status has not been investigated in the literature. However, some studies reported sex differences in mindfulness [22–24] and its subscales [24–26], and in their association with physical activity and dietary self-efficacy [26]; in addition, sex differences in the association between personality dimensions and BMI have been found [27].

There are several self-questionnaires aiming at assessing dispositional mindfulness, with a number of subscales ranging from 1 to 5. Mindfulness can be conceptualized as a “unified construct”, but multiple underlying factors have also been identified [8,22]. The Five Facet Mindfulness Questionnaire (FFMQ) was developed from several mindfulness questionnaires to assess an individual’s level of mindfulness in everyday life [28]. It has now been validated in different populations [24,25,28,29] and is widely used.

The aim of the present study was to explore the association between dispositional mindfulness and its facets, using the validated FFMQ, and weight status, in a large sample of participants in the NutriNet-Santé study. We also sought to determine whether these associations differ in men and women.

## Methods

### Study population

Participants were volunteers in the NutriNet-Santé study (https://www.etude-nutrinet-sante.fr), a large-scale population-based ongoing prospective observational cohort study that is exclusively web-based. It was launched in France in May 2009 with a scheduled follow-up of at least 10 years. It aims to investigate the relationship between nutrition and chronic disease risk, as well as determinants of dietary behavior and nutritional status. The study was implemented in the general French population (internet-using adult volunteers, age ≥18 years). The rationale, design and methodology of the study have been fully described elsewhere [30]. In brief, prior to inclusion, participants complete a baseline set of self-administered web-based questionnaires assessing dietary intake, physical activity, anthropometric characteristics, lifestyle, socioeconomic conditions and health status. As part of the follow-up, participants are requested to complete the same set of questionnaires every year. Moreover, each month, participants are invited by e-mail to fill in optional questionnaires related to dietary intake, determinants of eating behavior and nutritional and health status. This study is conducted in accordance with the Declaration of Helsinki, and all procedures were approved by the Institutional Review Board of the French Institute for Health and Medical Research (IRB Inserm n° 0000388FWA00005831) and the *Commission Nationale de l’Informatique et des Libertés* (CNIL n° 908450 and n° 909216). All participants provided informed consent with an electronic signature. This study is registered in EudraCT (n°2013-000929-31).

### Data collection

#### Mindfulness

Dispositional mindfulness was assessed in January 2013 using the French version [29] of the FFMQ [28]. The FFMQ assesses the propensity toward being mindful in daily life, and consists of 39 self-reported items covering five facets of mindfulness: “observing”, “describing”, “acting with awareness”, “non-judging” and “non-reactivity” [28]. The “observing” subscale includes noticing bodily sensations, emotions, odors and shapes of our surroundings; “describing” refers to labeling internal experiences with words; “acting with awareness” involves paying full attention to the activity of the moment, as opposed to behaving mechanically or inattentively; “non-judging” is related to acceptance and a non-judgmental approach to experiences; and “non-reactivity” refers to the tendency to allow thoughts and feelings to come and go without letting them take over. We slightly modified item 31 of the French version of the questionnaire by changing the word “pattern“, which is an Anglicism, into a French equivalent “contrastes”. We felt this change would improve the understanding of this statement in the general population. Items are rated on a 5-point Likert-type scale ranging from “never or very rarely true” to “very often or always true”. Individual item scores were summed in each of the five subscales, which were then summed into an overall mindfulness score. The resulting scores were divided by the number of items in each subscale or in the overall scale, as appropriate, leading to a possible range from 1 to 5. Higher scores indicated a greater degree of mindfulness. In our dataset, all items composing the overall mindfulness scale displayed good internal consistency (Cronbach’s α = 0.89) and subscale Cronbach’s α-coefficients ranged from 0.75 (“non-reactivity” subscale) to 0.89 (“describing” subscale).

#### Anthropometric measurements

Height and weight data were collected at enrollment and each year thereafter by a self-administered anthropometric questionnaire [31]. The closest available data to the FFMQ questionnaire were used. Average time between assessment of mindfulness and anthropometric measurements was 4.6 months (SD = 5.3).

BMI (kg/m^2^) was calculated as the ratio of weight to the square of height. Participants with BMI <25 were classified as underweight/normal weight, participants with 25 ≤ BMI < 30 were considered overweight (excluding obese) and participants with BMI ≥30 were considered obese in accordance with WHO reference values [32].

#### Covariate assessment

Potential covariates were identified based on evidence in the literature [8,26,33], i.e. age, education level, smoking status and physical activity. At inclusion, participants provided data on demographic, socio-economic and lifestyle characteristics, including sex, age, education level (primary, secondary or university), smoking status (never-smoker, former smoker or current smoker) and physical activity. Information was updated at one-year intervals. Physical activity was assessed using a short form of the French version of the International Physical Activity Questionnaire (IPAQ) [34]. The weekly energy expenditure expressed in metabolic equivalent task minutes per week was estimated and 3 categories of physical activity were defined [low (<30 min/d), moderate (30–59 min/d) and high (≥60 min/d)]. The practice of relaxation techniques was also assessed at the end of the questionnaire on mindfulness. Specifically, participants were asked whether they were currently using a relaxation technique such as yoga, tai-chi, qi-gong, sophrology, meditation or other. Sophrology is a European relaxation technique which relies on voluntary respiration, body relaxation, visualization of body parts and positive images associated with experiences. It is guided by the voice of a professional, but requires active involvement by the participant [35]. Participants who answered “yes” were considered current users, those who answered “yes, in the past” as former users and those with a negative answer as never-users. Current users were also asked about frequency and duration: “How often do you practice this(these) activity(ies)?” and “For how many years have you been doing in this(these) activity(ies)?” Participants who practiced at least once a week and for at least one year were considered regular users, while other participants were considered occasional users.

### Statistical analyses

Student’s *t* tests were used to compare included vs. excluded participants and to assess sex differences for continuous variables and chi-square tests for categorical variables. Quartiles of mindfulness and its subscale scores were defined for the entire sample and for each sex when required. Participant characteristics were compared across quartiles of mindfulness scores using linear contrast tests for continuous variables and Mantel-Haenszel chi-square tests for categorical variables. Multinomial logistic regression models were performed by calculating odds ratios (OR) and 95% CI to determine the strength of the association between weight status and the level of mindfulness and its subscales (taken in quartiles or continuous). Tests for linear trend were performed using the ordinal score on quartiles of mindfulness and its subscales scores. Interactions between mindfulness, its subscales and sex were tested. Since interactions between sex and mindfulness, as well as “describing” and “acting with awareness” subscales, were significant, all models were stratified by sex. Variables and interactions that reached P < 0.15 in univariate models were retained for inclusion in the initial multivariate model. All variables reached P<0.05 and were thus retained in the full model, including adjustment for age, education level, smoking status and physical activity. Missing covariate data for physical activity and education level were imputed using the multiple imputation method.

Sensitivity analyses were performed, excluding participants who reported current use of relaxation techniques, since previous studies had suggested that the “observing” subscale may operate differently in samples with and without meditation experience [8,28].

All tests of significance were two-sided and a p-value <0.05 was considered significant. All statistical analyses were performed using SAS software (version 9.3, SAS Institute Inc.).

## Results

### Characteristics of the sample

From the initial 116,023 participants who received the FFMQ, a total of 66,090 completed it. We excluded 2,400 pregnant women and 62 participants with missing data for weight or height, which left 63,628 participants available for analysis (49,228 women and 14,400 men). Compared to excluded participants, included participants were older (48.6 years for included participants vs. 41.6 years for excluded participants, *P*<0.0001), the proportion of men was higher (22.6 vs. 20.2%, *P*<0.0001), the proportion of individuals with university education level was higher (65.2 vs. 61.8%, *P*<0.0001), the proportion of smokers was lower (13.3 vs. 21.6%, *P*<0.0001) and the proportion of individuals with high physical activity level was higher (29.8 vs. 25.4%, *P*<0.0001). For included participants, the proportion of overweight persons (excluding obesity) was higher whereas the proportion of obese was lower compared with excluded participants (respectively 23.3 vs. 21.6 and 9.9 vs. 11.7 kg/m², *P*<0.0001).

Characteristics of the study population according to sex are shown in Table 1. Compared to men, women were younger, and percentages of never-smokers, former or current users of relaxation techniques and individuals with high education levels were higher for women, while the prevalence of individuals with high physical activity levels was lower. Women also had lower BMI, and the prevalence of overweight was lower than in men. Men showed slightly higher scores for mindfulness, “acting with awareness”, “non-judging” and “non-reactivity”, but slightly lower scores for “observing” and “describing” subscales.

**Table 1:** Individual characteristics of 63,628 participants in the NutriNet-Santé study (2013) according to sex.

	All	Women	Men	*P* ^a^
*n*	63,628	49,228	14,400	
Age (y)	48.6 ± 14.5^b^	47.1 ± 14.2	53.6 ± 14.4	<0.0001
Education level (%)				<0.0001
Primary	15.8	15.0	18.5	
Secondary	18.8	19.0	17.8	
University	65.2	65.7	63.4	
Missing data	0.3	0.4	0.3	
Smoking status (%)				<0.0001
Never-smoker	48.9	51.4	40.5	
Former smoker	37.8	34.9	47.9	
Current smoker	13.3	13.8	11.6	
Physical activity (%)				<0.0001
Low	20.9	21.5	18.9	
Moderate	36.5	37.7	32.4	
High	29.8	27.5	37.7	
Missing data	12.8	13.4	11.0	
Relaxation techniques (%)				<0.0001
Never-user	64.1	60.4	77.0	
Former user	18.2	20.5	10.6	
Occasional user	7.2	8.0	4.4	
Regular user	10.4	11.2	8.0	
BMI (kg/m²)	24.1 ± 4.6	23.8 ± 4.7	25.2 ± 3.8	<0.0001
Weight status (%)				<0.0001
Underweight/normal weight (<25 kg/m²)	66.8	70.4	54.6	
Overweight (25–29.99 kg/m²)	23.3	19.8	35.1	
Obesity (≥30 kg/m²)	9.9	9.8	10.2	
Mindfulness (1–5)^c^	3.32 ± 0.43	3.31 ± 0.43	3.37 ± 0.40	<0.0001
Observing (1–5) ^c^	3.44 ± 0.66	3.47 ± 0.65	3.33 ± 0.68	<0.0001
Describing (1–5) ^c^	3.28 ± 0.74	3.29 ± 0.75	3.23 ± 0.71	<0.0001
Acting with awareness (1–5) ^c^	3.59 ± 0.69	3.56 ± 0.69	3.70 ± 0.68	<0.0001
Non-judging (1–5) ^c^	3.41 ± 0.73	3.37 ± 0.73	3.53 ± 0.71	<0.0001
Non-reactivity (1–5) ^c^	2.82 ± 0.59	2.77 ± 0.58	3.01 ± 0.57	<0.0001

Abbreviation: BMI, body mass index.

^a^Student *t* test or Pearson’s chi^2^-test as appropriate.

^b^Mean ± SD (all such values).

^c^Score range.

### Socio-demographic and lifestyle correlates of mindfulness

Socio-demographic and lifestyle characteristics across quartiles of mindfulness scores, stratified by sex, are shown in Table 2.

**Table 2:** Individual characteristics of 63,628 participants in the NutriNet-Santé study (2013) according to mindfulness quartiles and sex.

	Women (n = 49,228)						Men (n = 14,400)					
	Q1 (n = 12,251)	Q2 (n = 11,956)	Q3 (n = 12,971)	Q4 (n = 12,050)	F or Chi^2^ values^a^	*P* ^b^	Q1 (n = 3,469)	Q2 (n = 3,851)	Q3 (n = 3,487)	Q4 (n = 3,593)	F or Chi^2^ values^a^	*P* ^b^
Age (y)	45.3 ± 14.5 ^c^	46.1 ± 14.4	47.3 ± 14.1	49.7 ± 13.3	611.3	<.0001	52.1 ± 15.0	53.6 ± 14.6	53.9 ± 14.3	54.8 ± 13.7	61.9	<.0001
Educational level (%)					495.7	<.0001					174.2	<.0001
Primary	18.4	16.8	14.1	10.5			23.3	20.1	17.0	13.6		
Secondary	21.3	19.5	18.5	16.8			19.5	18.6	17.9	15.3		
University	60.0	63.3	67.0	72.4			56.9	61.0	64.8	70.8		
Missing data	0.3	0.4	0.4	0.4			0.2	0.2	0.3	0.3		
Smoking status (%)					15.4	<.0001					14.1	0.0002
Never-smoker	52.4	52.0	51.9	49.0			42.4	40.6	40.1	39.0		
Former smoker	33.8	34.3	34.4	37.0			47.0	48.7	47.4	48.3		
Current smoker	13.7	13.7	13.7	14.1			10.6	10.7	12.6	12.7		
Physical activity (%)					488.8	<.0001					118.2	<.0001
Low	25.7	22.1	20.5	17.5			22.8	19.4	17.9	15.6		
Moderate	36.6	37.5	38.2	38.5			32.3	32.3	32.4	32.7		
High	22.5	26.0	28.4	33.1			32.7	36.1	38.9	43.1		
Missing data	15.1	14.4	12.9	10.9			12.3	12.3	10.9	8.6		
Relaxation techniques (%)					1128.6	<.0001					244.2	<.0001
Nerver-user	65.5	64.4	61.1	50.3			81.0	80.0	77.9	69.2		
Former user	20.8	19.9	20.1	21.2			10.6	10.3	10.4	11.1		
Occasional user	7.5	7.9	7.8	9.0			4.3	3.7	4.2	5.4		
Regular user	6.2	7.9	11.0	19.6			4.2	6.0	7.6	14.3		
BMI (kg/m²)	24.1 ± 5.1	23.8 ± 4.7	23.7 ± 4.6	23.5 ± 4.5	102.8	<.0001	25.3 ± 4.1	25.2 ± 3.8	25.2 ± 3.8	25.2 ± 3.6	3.7	0.054
Weight status (%)					98.4	<.0001					5.3	0.021
Normal weight (<25 kg/m²)	67.8	70.0	71.0	72.8			53.7	55.2	54.4	55.19		
Overweight (25–29.99 kg/m²)	20.5	20.1	19.8	18.9			34.5	34.8	35.6	35.71		
Obese (≥30 kg/m²)	11.7	9.9	9.2	8.4			11.8	10.0	10.0	9.10		

Abbreviation: BMI, body mass index.

^a^ F value for analysis of variance (linear contrast tests) and Chi^2^ value for Mantel-Haenszel tests.

^b^ On the basis of linear contrast tests (continuous variables) or Mantel-Haenszel tests (categorical variables).

^c^Mean ± SD (all such values).

Men and women with higher mindfulness scores showed greater physical activity, a higher education level, practiced relaxation techniques more often, were older, and were slightly more often former smokers, than participants with lower mindfulness scores. In addition, women with higher mindfulness scores were less often overweight or obese, and had a slightly lower BMI, while men with higher mindfulness scores were less often obese. Finally, bivariate correlations between total mindfulness and BMI were: r = -0.05 (p<0.0001) for women and r = -0.02 (p<0.05) for men, while between total mindfulness and age they were r = 0.12 (p<0.0001) for women and r = 0.07 (p<0.0001) for men. See S1 Table.

### Association of mindfulness and its subscales with overweight and obesity according to sex

Analysis of the association between mindfulness score, taken in quartiles and continuous, and weight status showed similar results (Table 3). After adjustment for socio-demographic and lifestyle confounding factors, women with higher overall mindfulness scores were less likely to be overweight and even less likely obese. All subscales were inversely associated with overweight and obesity except for a non-significant association between overweight and “non-judging”. In addition, the strongest association was found for the “observing” subscale. In contrast, in men, there was no significant association between overall mindfulness and overweight. Higher overall mindfulness was significantly associated with lower odds of obesity; however, the OR corresponding to Q3 vs. Q1 was not significant, indicating the absence of a linear relationship. Both “observing” and “non-reactivity” subscales were inversely associated with overweight and obesity. However, no association was found for “describing” and “non-judging” subscales. Finally, a positive association was observed between “acting with awareness” and overweight (both the trend across quartiles and the continuous score), but none of the quartiles vs. Q1 were significant and no association was found for obesity.

**Table 3:** Associations between mindfulness scores and overweight (excluding obesity) and obesity according to sex in 63,628 participants (NutriNet-Santé study, 2013)^a^,^b^.

	Q1	Q2	Q3	Q4		Continuous	
		OR (95% CI)	OR (95% CI)	OR (95% CI)	*P trend*	OR (95% CI)	*P*
**Women (n = 49,228)**							
Mindfulness							
Overweight^c^	ref	0.95 (0.89, 1.01)	0.92 (0.86, 0.98)	0.84 (0.79, 0.90)	<0.0001	0.85 (0.80, 0.89)	<0.0001
Obese^d^	ref	0.85 (0.78, 0.92)	0.79 (0.72, 0.86)	0.71 (0.65, 0.78)	<0.0001	0.71 (0.66, 0.77)	<0.0001
Observing							
Overweight^c^	ref	0.95 (0.89, 1.01)	0.91 (0.85, 0.97)	0.79 (0.74, 0.84)	<0.0001	0.87 (0.84, 0.90)	<0.0001
Obese^d^	ref	0.88 (0.81, 0.96)	0.83 (0.76, 0.91)	0.70 (0.64, 0.77)	<0.0001	0.82 (0.79, 0.86)	<0.0001
Describing							
Overweight^c^	ref	0.97 (0.90, 1.03)	0.97 (0.91, 1.04)	0.90 (0.84, 0.96)	0.0032	0.95 (0.92, 0.98)	0.0006
Obese^d^	ref	0.93 (0.85, 1.01)	0.82 (0.75, 0.90)	0.83 (0.76, 0.91)	<0.0001	0.90 (0.86, 0.94)	<0.0001
Acting with awareness							
Overweight^c^	ref	0.91 (0.85, 0.97)	0.94 (0.88, 1.00)	0.92 (0.86, 0.99)	0.048	0.95 (0.92, 0.98)	0.0039
Obese^d^	ref	0.85 (0.78, 0.93)	0.86 (0.79, 0.93)	0.86 (0.78, 0.94)	0.001	0.91 (0.87, 0.95)	<0.0001
Non-judging							
Overweight^c^	ref	1.00 (0.94, 1.07)	1.00 (0.94, 1.07)	0.95 (0.89, 1.02)	0.21	0.98 (0.95, 1.01)	0.16
Obese^d^	ref	0.84 (0.77, 0.92)	0.85 (0.78, 0.92)	0.78 (0.72, 0.86)	<0.0001	0.88 (0.84, 0.92)	<0.0001
Non-reactivity							
Overweight^c^	ref	1.03 (0.97, 1.10)	1.01 (0.94, 1.08)	0.90 (0.84, 0.96)	0.0002	0.92 (0.88, 0.95)	<0.0001
Obese^d^	ref	0.85 (0.78, 0.93)	0.82 (0.75, 0.90)	0.77 (0.71, 0.84)	<0.0001	0.84 (0.80, 0.89)	<0.0001
**Men (n = 14,400)**							
Mindfulness							
Overweight^c^	ref	0.95 (0.86, 1.05)	1.00 (0.90, 1.11)	0.98 (0.88, 1.09)	0.96	0.98 (0.89, 1.07)	0.64
Obese^d^	ref	0.83 (0.71, 0.97)	0.88 (0.75, 1.03)	0.81 (0.69, 0.96)	0.034	0.81 (0.70, 0.94)	0.0063
Observing							
Overweight^c^	ref	0.95 (0.85, 1.06)	0.88 (0.80, 0.97)	0.86 (0.78, 0.95)	0.0011	0.90 (0.86, 0.95)	0.0002
Obese^d^	ref	0.86 (0.73, 1.01)	0.80 (0.68, 0.93)	0.75 (0.64, 0.89)	0.0003	0.86 (0.79, 0.93)	0.0003
Describing							
Overweight^c^	ref	1.00 (0.90, 1.12)	1.07 (0.97, 1.18)	1.07 (0.96, 1.19)	0.12	1.05 (0.99, 1.10)	0.088
Obese^d^	ref	0.88 (0.75, 1.04)	1.02 (0.87, 1.19)	1.02 (0.86, 1.20)	0.47	1.02 (0.94, 1.10)	0.69
Acting with awareness							
Overweight^c^	ref	0.92 (0.82, 1.02)	1.04 (0.94, 1.15)	1.11 (1.00, 1.24)	0.0068	1.09 (1.03, 1.15)	0.0015
Obese^d^	ref	0.81 (0.68, 0.96)	1.02 (0.87, 1.20)	1.01 (0.86, 1.19)	0.28	0.99 (0.91, 1.08)	0.90
Non-judging							
Overweight^c^	ref	0.98 (0.89, 1.09)	1.02 (0.92, 1.12)	0.98 (0.89, 1.09)	0.94	0.99 (0.94, 1.04)	0.73
Obese^d^	ref	0.92 (0.79, 1.08)	1.00 (0.86, 1.17)	0.93 (0.79, 1.09)	0.59	0.98 (0.90, 1.06)	0.61
Non-reactivity							
Overweight^c^	ref	0.92 (0.83, 1.02)	0.89 (0.80, 1.00)	0.85 (0.76, 0.94)	0.0014	0.90 (0.85, 0.96)	0.0022
Obese^d^	ref	0.71 (0.61, 0.82)	0.68 (0.57, 0.81)	0.69 (0.59, 0.81)	<0.0001	0.77 (0.69, 0.85)	<0.0001

Abbreviations: Q, Quartile; OR, Odds Ratio; 95% CI, 95% Confidence Interval

^a^Adjusted for age, education level, smoking status and physical activity.

^b^Underweight/normal weight as reference

^c^(25–29.99 kg/m²)

^d^(≥30 kg/m²)

In sensitivity analyses, exclusion of current users of relaxation techniques did not change results, apart from the facet “acting with awareness”, for which the OR corresponding to Q4 vs. 1 for overweight became significant in men (OR = 1.15 [1.02–1.29]).

## Discussion

This is the first general population-based study to examine relationships between dispositional mindfulness, its facets and weight status. In women, greater overall mindfulness was associated with lower odds of being overweight, and to an even greater extent, obese. Overall, all subscales were associated with weight status, with the strongest association found for the “observing” subscale. In contrast, in men, higher mindfulness was associated with lower odds of obesity only, and only the “observing” and “non-reactivity” subscales were inversely associated with weight status.

### Sex-specific level of mindfulness

In this large nationwide sample, absolute scores of dispositional mindfulness and its subscales were within the same range as in previous studies [8,25,29,36]. In our study, men had slightly greater scores of overall mindfulness than did women. The few studies assessing sex differences using measures of mindfulness based on different concepts contained contrasting results [22,23,37]. In agreement with our study, men had higher scores of overall mindfulness than women using the Comprehensive Inventory of Mindfulness Experiences Beta [22] and the Cognitive and Affective Mindfulness-Scale-Revised [23], whereas another study reported no differences between men and women using the Freiburg Mindfulness Inventory [37]. Specifically, in our study, men had slightly higher scores of “acting with awareness”, “non-judging” and “non-reactivity” and lower scores of “observing” and “describing” compared with women. Similarly, previous studies using the FFMQ showed that men had significantly higher scores for the “non-reactivity” facet [25,26] and significantly lower scores for the “observing” [24,26] and “describing” facets [24]. However, other studies showed no sex-specific differences [8,38]. These sex differences in the mindfulness scale and subscales were small, although statistically significant due to the large sample size, while within-sex differences were much higher than between-sex differences.

### Overall mindfulness and weight status

In women, a higher mindfulness score was associated with lower odds of overweight and, to a greater extent, obesity, while this inverse association was observed for obesity but not overweight in men. In addition, contrary to women, the association did not seem linear in men. The observed moderating effect of sex can be set against stronger associations found in women between unhealthy eating behavior, including emotional eating and overweight [39]. In the literature, mindfulness was inversely associated with weight gain in male military recruits [9], and positively with weight loss in students [4]. In addition, college students with varying mindfulness levels showed no differences in anthropometric measurements [10]. Consequently, our findings in a general population greatly expand current knowledge gained in previous observational studies which were carried out on small samples of specific groups of individuals, and which did not take into account confounding factors. Mindfulness-based interventions have been shown to help overweight/obese participants maintain [17] and reduce weight [18,19], as well as military recruits [40] and students seeking to lose weight [4]. However, other studies observed non-significant results [20,21], and the very few randomized controlled trials with active controls provided only low evidence of no effect on weight [13,41,42]. In addition, most included very small or homogeneous samples [4,17–21,40–42]. Finally, observational studies focusing on mindful eating, which describes non-judgmental awareness of physical or emotional sensations associated with eating, have also shown that this specific dimension has a negative association with BMI, in agreement with our data [11,12].

Several hypotheses might explain why dispositional mindfulness is associated with weight. Mindfulness may enhance self-regulation [43,44], including that of appetite and consequently, energy balance and weight control. Participants with higher mindfulness scores have been found to report smaller serving sizes of energy-dense foods [36]. Another hypothesis is that mindfulness reduces eating driven by emotional or external cues. Consistent with this notion, negative associations of mindfulness scores with emotional and uncontrolled eating have been reported [45]. Mindfulness-based interventions have also been shown to decrease emotional eating and eating triggered by external cues [16], as well as food craving [21] and binge eating [41]. Mindfulness intervention has also been shown to reduce chronic stress [17], which could, in turn, reduce abdominal adiposity. Overall, these findings suggest that mindfulness minimizes automatic and emotional responses to food and in the eating process [20,44]. However, we cannot exclude reverse causality. Weight changes could also modify levels of mindfulness or specific aspects of it. For example, weight gain might have a negative impact on self-acceptance [46], which in turn may lead to lower levels of “non-judging”.

### Facets of mindfulness and weight status

“Observing” was inversely associated with overweight and obesity in both men and women. Yet mindfulness begins by observing and attending to one’s moment-to-moment internal and external experiences [47]. It is therefore a core aspect of mindfulness that is included in contemporary operational definitions [44,48]. “Observing” has been shown to be associated with healthy behavior, including higher fruit and vegetable intake, and reported self-efficacy in reducing calories in both men and women [26].

In our study, “describing”, “non-judging” and “acting with awareness” were inversely associated with overweight and obesity in women only, except for the “non-judging” subscale that was not associated with overweight. This is in agreement with a previous study that found that “describing” was associated with physical activity, self-efficacy at resisting dietary relapse, and self-efficacy at reducing fat intake, but in women only [26]. Mood and emotional regulation differ between men and women [49,50] and these differences could potentially explain existing differential associations for the three facets. Specifically, the “non-judging” component of mindfulness may allow women to accept their appearance and their thoughts rather than attempting to suppress them. Women, to a greater extent than men, have been shown to rely on avoidance techniques, including food-thought suppression [51]. Paradoxically, however, attempting to avoid unwanted thoughts about eating or weight has been shown not only to increase the frequency of these thoughts [52], but also to exacerbate food-seeking behavior [53]. Conversely, dispositional mindfulness is negatively correlated with experiential avoidance, thought suppression [23] and habitual negative thinking [54]. People who present high levels of dispositional mindfulness have an inherent ability to observe their thoughts as transient mental events, in a decentralized way [55]. A recent experimental study showed that activation of this skill prevented hunger from enhancing the attractiveness of unhealthy foods, resulting in healthier food choices in both laboratory and real life conditions [55]. Acceptance-based craving intervention in an overweight or obese adult population has also proven useful for reducing obsessive thoughts about food and eating [20].

### Strengths and limitations

One strength of our study was its large sample size, providing high statistical power. The use of the internet for data collection gave access to a vast heterogeneous sample of volunteers in whom a wide range of socio-demographic and lifestyle characteristics were assessed, so as to effectively control for potential confounding factors [30] and improve the meaningfulness of the effects detected. The FFMQ is a useful instrument for measuring mindfulness that has been widely used and translated into several languages, including French [29]. It has satisfactory internal consistency, replicated in the present study.

The main limitation of the study was its cross-sectional design, preventing inference of causality. Prevalence of overweight was estimated using self-reported anthropometric data and may have led to misclassification. However, standardized clinical measurements on a subsample (n = 2513) of the cohort confirmed the validity of the web-based self-reported heights and weights from the NutriNet-Santé study and the resulting BMI classes [56]. Caution is also needed when generalizing our results, since the NutriNet-Santé study is a long-term cohort and participants are recruited on a voluntary basis, implying that they might have increased health consciousness and interest in nutritional issues. A selection bias might also have occurred, given the large sample loss, due to the fact that the questionnaire was optional. Finally, the sample size can also be a constraint since it produces significant results even though differences are small but it enables highly accurate estimates. To assess the significance of our results from a public health perspective, we compared odds related to the “observing” dimension in women with those linked to education level, which is a well-known determinant of obesity [57,58]. In our study, the OR for obesity comparing university to primary education level was 0.41 [0.37–0.44]. Thus, an OR of 0.70 [0.64–0.77] for obesity comparing Q4 to Q1 of “observing” scores is probably meaningful at a population level.

## Conclusion

The present cross-sectional study provides the first data on dispositional mindfulness in relation to overweight and obesity in a large population-based sample. In women, greater overall mindfulness was associated with lower risk of overweight and obesity. Overall, all subscales were inversely associated with weight status. In contrast, in men, higher mindfulness was associated only with lower risk of obesity, and only the “observing” and “non-reactivity” subscales were associated with lower risk of overweight and obesity. These preliminary findings support the interest of a shift in perspective taking into account positive psychological and cognitive factors such as dispositional mindfulness in the investigation of obesity and its associated factors. More studies, and in particular, longitudinal studies for identifying causality, are necessary to confirm and further refine these findings.

## Supporting Information

S1 Table Pearson bivariate correlations among FFMQ scores, BMI, and age according to sex in 63,628 participants (NutriNet-Santé study, 2013). (DOCX)

## References

[1] World Health Organization (2003) Diet, Nutrition and the Prevention of Chronic Diseases Geneva: World Health Organization Technical Report Series 916. 12768890

[2] Robinson E, Aveyard P, Daley A, Jolly K, Lewis A, Lycett D et al (2013) Eating attentively: a systematic review and meta-analysis of the effect of food intake memory and awareness on eating. Am J Clin Nutr 97:728–42. 10.3945/ajcn.112.045245 23446890 PMC3607652

[3] Liebman M, Pelican S, Moore SA, Holmes B, Wardlaw MK, Melcher LM et al (2003) Dietary intake, eating behavior, and physical activity-related determinants of high body mass index in rural communities in Wyoming, Montana, and Idaho. Int J Obes 27:684–92. 12833112 10.1038/sj.ijo.0802277

[4] Mantzios M, Wilson JC (2014) Making concrete construals mindful: a novel approach for developing mindfulness and self-compassion to assist weight loss. Psychol Health 29:422–41. 10.1080/08870446.2013.863883 24215123

[5] Jeffery RW, Drewnowski A, Epstein LH, Stunkard AJ, Wilson GT, Wing RR et al (2000) Long-term maintenance of weight loss: current status. Health Psychol 19:5–16. 10709944 10.1037/0278-6133.19.suppl1.5

[6] Mann T, Tomiyama AJ, Westling E, Lew AM, Samuels B, Chatman J (2007) Medicare's search for effective obesity treatments: diets are not the answer. Am Psychol 62:220–33. 17469900 10.1037/0003-066X.62.3.220

[7] Kabat-Zinn J (2003) Mindfulness-Based Interventions in Context: Past, Present, and Future. Clinical Psychology: Science and Practice 10:144–56.

[8] Baer RA, Smith GT, Lykins E, Button D, Krietemeyer J, Sauer S et al (2008) Construct validity of the five facet mindfulness questionnaire in meditating and nonmeditating samples. Assessment 15:329–42. 10.1177/1073191107313003 18310597

[9] Mantzios M, Wilson JC, Linnell M, Morris P (2014) The role of negative cognition, intolerance onf uncertainty, mindfulness, and self-compassion in weight regulation among male army recruits. Mindfulness 1–8 10.1007/s12671-014-0286-2 25126133

[10] Grinnell S, Greene G, Melanson K, Blissmer B, Lofgren IE (2011) Anthropometric and behavioral measures related to mindfulness in college students. J Am Coll Health 59:539–45. 10.1080/07448481.2011.555932 21660809

[11] Framson C, Kristal AR, Schenk JM, Littman AJ, Zeliadt S, Benitez D (2009) Development and validation of the mindful eating questionnaire. J Am Diet Assoc 109:1439–44. 10.1016/j.jada.2009.05.006 19631053 PMC2734460

[12] Moor KR, Scott AJ, McIntosh WD (2013) Mindful Eating and Its Relationship to Body Mass Index and Physical Activity Among University Students. Mindfulness 4:269–74.

[13] Goyal M, Singh S, Sibinga EM, Gould NF, Rowland-Seymour A, Sharma R et al (2014) Meditation programs for psychological stress and well-being: a systematic review and meta-analysis. JAMA Intern Med 174:357–68. 10.1001/jamainternmed.2013.13018 24395196 PMC4142584

[14] Grossman P, Niemann L, Schmidt S, Walach H (2004) Mindfulness-based stress reduction and health benefits. A meta-analysis. J Psychosom Res 57:35–43. 15256293 10.1016/S0022-3999(03)00573-7

[15] Godsey J (2013) The role of mindfulness based interventions in the treatment of obesity and eating disorders: an integrative review. Complement Ther Med 21:430–9. 10.1016/j.ctim.2013.06.003 23876574

[16] O'Reilly GA, Cook L, Spruijt-Metz D, Black DS (2014) Mindfulness-based interventions for obesity-related eating behaviours: a literature review. Obes Rev 15:453–61. 10.1111/obr.12156 24636206 PMC4046117

[17] Daubenmier J, Kristeller J, Hecht FM, Maninger N, Kuwata M, Jhaveri K et al (2011) Mindfulness Intervention for Stress Eating to Reduce Cortisol and Abdominal Fat among Overweight and Obese Women: An Exploratory Randomized Controlled Study. J Obes 2011:651936 10.1155/2011/651936 21977314 PMC3184496

[18] Tapper K, Shaw C, Ilsley J, Hill AJ, Bond FW, Moore L (2009) Exploratory randomised controlled trial of a mindfulness-based weight loss intervention for women. Appetite 52:396–404. 10.1016/j.appet.2008.11.012 19101598

[19] Timmerman GM, Brown A (2012) The effect of a mindful restaurant eating intervention on weight management in women. J Nutr Educ Behav 44:22–8. 10.1016/j.jneb.2011.03.143 22243980 PMC3259454

[20] Alberts HJ, Mulkens S, Smeets M, Thewissen R (2010) Coping with food cravings. Investigating the potential of a mindfulness-based intervention. Appetite 55:160–3. 10.1016/j.appet.2010.05.044 20493913

[21] Alberts HJ, Thewissen R, Raes L (2012) Dealing with problematic eating behaviour. The effects of a mindfulness-based intervention on eating behaviour, food cravings, dichotomous thinking and body image concern. Appetite 58:847–51. 10.1016/j.appet.2012.01.009 22265753

[22] Bergomi C, Tschacher W, Kupper Z (2012) Measuring Mindfulness: First Steps Towards the Development of a Comprehensive Mindfulness Scale. Mindfulness 4:18–32.

[23] Feldman G, Hayes A, Kumar S, Greeson J, Laurenceau J-P (2007) Mindfulness and Emotion Regulation: The Development and Initial Validation of the Cognitive and Affective Mindfulness Scale-Revised (CAMS-R). Journal of Psychopathology and Behavioral Assessment 29:177–90.

[24] Lilja JL, Frodi-Lundgren A, Hanse JJ, Josefsson T, Lundh LG, Skold C et al (2011) Five Facets Mindfulness Questionnaire—reliability and factor structure: a Swedish version. Cogn Behav Ther 40:291–303. 10.1080/16506073.2011.580367 21770845

[25] Dundas I, Vollestad J, Binder PE, Sivertsen B (2013) The Five Factor Mindfulness Questionnaire in Norway. Scand J Psychol 54:250–60. 10.1111/sjop.12044 23480438

[26] Gilbert D, Waltz J (2010) Mindfulness and Health Behaviors. Mindfulness 1:227–34.

[27] Faith MS, Flint J, Fairburn CG, Goodwin GM, Allison DB (2001) Gender differences in the relationship between personality dimensions and relative body weight. Obes Res 9:647–50. 11595783 10.1038/oby.2001.86

[28] Baer RA, Smith GT, Hopkins J, Krietemeyer J, Toney L (2006) Using self-report assessment methods to explore facets of mindfulness. Assessment 13:27–45. 16443717 10.1177/1073191105283504

[29] Heeren A, Douilliez C, Peschard V, Debrauwere L, Philippot P (2011) Cross-cultural validity of the Five Facets Mindfulness Questionnaire: Adaptation and validation in a French-speaking sample. Eur Rev Appl Psychol 61:147–151.

[30] Hercberg S, Castetbon K, Czernichow S, Malon A, Mejean C, Kesse E et al (2010) The Nutrinet-Sante Study: a web-based prospective study on the relationship between nutrition and health and determinants of dietary patterns and nutritional status. BMC Public Health 10:242 10.1186/1471-2458-10-242 20459807 PMC2881098

[31] Touvier M, Mejean C, Kesse-Guyot E, Pollet C, Malon A, Castetbon K et al (2010) Comparison between web-based and paper versions of a self-administered anthropometric questionnaire. Eur J Epidemiol 25:287–96. 10.1007/s10654-010-9433-9 20191377

[32] World Health Organization (2000) Obesity: preventing and managing the global epidemic. report of a WHO consultation Geneva: World Health Organization Technical Report Series 894. 11234459

[33] Roberts KC, Danoff-Burg S (2010) Mindfulness and health behaviors: is paying attention good for you? J Am Coll Health 59:165–73. 10.1080/07448481.2010.484452 21186446

[34] Craig CL, Marshall AL, Sjostrom M, Bauman AE, Booth ML, Ainsworth BE et al (2003) International physical activity questionnaire: 12-country reliability and validity. Med Sci Sports Exerc 35:1381–95. 12900694 10.1249/01.MSS.0000078924.61453.FB

[35] Fiorletta P (2010) Fondements et théories de la Sophrologie Caycédienne [*Theoretical basis of Professor Caycedo's sophrology]* . Kinesither rev 103:24–30.

[36] Beshara M, Hutchinson AD, Wilson C (2013) Does mindfulness matter? Everyday mindfulness, mindful eating and self-reported serving size of energy dense foods among a sample of South Australian adults. Appetite 67:25–9. 10.1016/j.appet.2013.03.012 23548262

[37] Trousselard M, Steiler D, Raphel C, Cian C, Duymedjian R, Claverie D et al (2010) Validation of a French version of the Freiburg Mindfulness Inventory—short version: relationships between mindfulness and stress in an adult population. Biopsychosoc Med 4:8 10.1186/1751-0759-4-8 20704696 PMC2927476

[38] Baer RA, Smith GT, Allen KB (2004) Assessment of mindfulness by self-report: the Kentucky inventory of mindfulness skills. Assessment 11:191–206. 15358875 10.1177/1073191104268029

[39] Péneau S, Menard E, Méjean C, Bellisle F, Hercberg S (2013) Sex and dieting modify the association between emotional eating and weight status. Am J Clin Nutr 97:1307–13. 10.3945/ajcn.112.054916 23576047

[40] Mantzios M, Wilson JC (2014) Exploring mindfulness and mindfulness with self-compassion-centered interventions to assist weight loss: theoretical considerations and preliminary results of a randomized pilot study. Mindfulness 1–12 10.1007/s12671-014-0325-z 25126133

[41] Kristeller J, Wolever RQ, Sheets V (2013) Mindfulness-Based Eating Awareness Training (MB-EAT) for Binge Eating: A Randomized Clinical Trial. Mindfulness 3:1–16.

[42] Miller CK, Kristeller JL, Headings A, Nagaraja H, Miser WF (2012) Comparative effectiveness of a mindful eating intervention to a diabetes self-management intervention among adults with type 2 diabetes: a pilot study. J Acad Nutr Diet 112:1835–42. 10.1016/j.jand.2012.07.036 23102183 PMC3485681

[43] Kristeller JL, Wolever RQ (2011) Mindfulness-based eating awareness training for treating binge eating disorder: the conceptual foundation. Eat Disord 19:49–61. 10.1080/10640266.2011.533605 21181579

[44] Shapiro SL, Carlson LE, Astin JA, Freedman B (2006) Mechanisms of mindfulness. J Clin Psychol 62:373–86. 16385481 10.1002/jclp.20237

[45] Lattimore P, Fisher N, Malinowski P (2011) A cross-sectional investigation of trait disinhibition and its association with mindfulness and impulsivity. Appetite 56:241–8. 10.1016/j.appet.2010.12.007 21146571

[46] Carr D, Jaffe K (2012) The psychological consequences of weight change trajectories: evidence from quantitative and qualitative data. Econ Hum Biol 10:419–30. 10.1016/j.ehb.2012.04.007 22580044 PMC3414673

[47] Kabat-Zinn J (1990) Full catastrophe living: Using the wisdom of your mind to face stress, pain and illness. New York: Delta Trade Paperbacks.

[48] Bishop SR, Lau M, Shapiro S, Carlson L, Anderson ND, Carmody J et al (2004) Mindfulness: A proposed Operational Definition. Clinical Psychology: Science and Practice 11:230–41.

[49] Gardener EK, Carr AR, Macgregor A, Felmingham KL (2013) Sex differences and emotion regulation: an event-related potential study. PLoS One 8:e73475 10.1371/journal.pone.0073475 24204562 PMC3813629

[50] Thayer RE, Newman JR, McClain TM (1994) Self-regulation of mood: strategies for changing a bad mood, raising energy, and reducing tension. J Pers Soc Psychol 67:910–25. 7983582 10.1037//0022-3514.67.5.910

[51] Barnes RD, Tantleff-Dunn S (2010) A preliminary investigation of sex differences and the mediational role of food thought suppression in the relationship between stress and weight cycling. Eat Weight Disord 15:e265–e269. 21406950 10.1007/BF03325308

[52] Abramowitz JS, Tolin DF, Street GP (2001) Paradoxical effects of thought suppression: a meta-analysis of controlled studies. Clin Psychol Rev 21:683–703. 11434226 10.1016/s0272-7358(00)00057-x

[53] Johnston L, Bulik CM, Anstiss V (1999) Suppressing thoughts about chocolate. Int J Eat Disord 26:21–7. 10349580 10.1002/(sici)1098-108x(199907)26:1<21::aid-eat3>3.0.co;2-7

[54] Verplanken B, Friborg O, Wang CE, Trafimow D, Woolf K (2007) Mental habits: metacognitive reflection on negative self-thinking. J Pers Soc Psychol 92:526–41. 17352607 10.1037/0022-3514.92.3.526

[55] Papies EK, Pronk TM, Keesman M, Barsalou LW (2015) The benefits of simply observing: Mindful attention modulates the link between motivation and behavior. J Pers Soc Psychol 108:148–70. 10.1037/a0038032 25347126

[56] Lassale C, Péneau S, Touvier M, Julia C, Galan P, Hercberg S et al (2013) Validity of web-based self-reported weight and height: results of the Nutrinet-Sante study. J Med Internet Res 15:e152 10.2196/jmir.2575 23928492 PMC3742400

[57] Lantz PM, House JS, Lepkowski JM, Williams DR, Mero RP, Chen J (1998) Socioeconomic factors, health behaviors, and mortality: results from a nationally representative prospective study of US adults. JAMA 279:1703–8. 9624022 10.1001/jama.279.21.1703

[58] Molarius A, Seidell JC, Sans S, Tuomilehto J, Kuulasmaa K (2000) Educational level, relative body weight, and changes in their association over 10 years: an international perspective from the WHO MONICA Project. Am J Public Health 90:1260–8. 10937007 10.2105/ajph.90.8.1260 PMC1446346

