# Supplementary material for: Association between Mindfulness and Weight Status in a General Population from the NutriNet-Santé Study
Source: PLoS One. 2015 Jun 3;10(6):e0127447. doi: 10.1371/journal.pone.0127447 (PMC4454654; doi:10.1371/journal.pone.0127447)
Supplement: S1 Table — (DOCX) [file pone.0127447.s001.docx]

**Table S1**: Pearson bivariate correlations among FFMQ scores, BMI, and age according to sex in 63,628 participants (NutriNet-Santé study, 2013)^a^

|  | Mindfulness | Observing | Describing | Acting with awareness | Non-judging | Non-reactivity | BMI | | Age | |  |
| --- | --- | --- | --- | --- | --- | --- | --- | --- | --- | --- | --- |
| Mindfulness |  | 0.54*** | 0.73*** | 0.65*** | 0.50*** | 0.51*** | -0.02* | | 0.07*** | |  |
| Observing | 0.57*** |  | 0.42*** | 0.06*** | -0.17*** | 0.28*** | -0.03*** | | 0.04*** | |  |
| Describing | 0.73*** | 0.43*** |  | 0.32*** | 0.11*** | 0.25*** | 0.01 | | 0.05*** | |  |
| Acting with awareness | 0.67*** | 0.14*** | 0.33*** |  | 0.38*** | 0.08*** | 0.02** | | 0.10*** | |  |
| Non-judging | 0.61*** | -0.01** | 0.21*** | 0.39*** |  | 0.10*** | -0.01 | | 0.02* | |  |
| Non-reactivity | 0.58*** | 0.28*** | 0.28*** | 0.19*** | 0.27*** |  | -0.06*** | | -0.02 | |  |
| BMI | -0.05*** | -0.05*** | -0.05*** | -0.02*** | -0.02*** | -0.03*** | |  | | 0.23*** | |
| Age | 0.12*** | 0.13*** | 0.04*** | 0.06*** | 0.04*** | 0.11*** | 0.17*** | |  | |  |

Abbreviation: BMI, body mass index.

^a^Values for men are presented above the diagonal whereas values for women are presented below the diagonal. **P*<0.05; ***P*<0.01; ****P*<0.0001
